# A225 QUANTIFICATION OF BONE DENSITY AND DIETARY RISK FACTORS FOR BONE FRAGILITY IN INFLAMMATORY BOWEL DISEASE

**DOI:** 10.1093/jcag/gwac036.225

**Published:** 2023-03-07

**Authors:** A Macci, R Klassen, R Rosentreter, J Szostakiwskyj, E Billington, R Panaccione, M Raman, L Burt, C Lu

**Affiliations:** 1 Department of Medicine , University of Calgary Cumming School of Medicine; 2 Department of Medicine , Alberta Health Services; 3 Division of Gastroenterology, Department of Medicine; 4 Department of Radiology, University of Calgary Cumming School of Medicine , Calgary, Canada

## Abstract

**Background:**

Nutrition and bone fracture risk are intimately related in inflammatory bowel disease (IBD). Crohn’s disease (CD) patients are at increased risk of low bone mineral density (BMD) and fractures. This may be due to chronic inflammation, corticosteroid exposure, inadequate consumption of nutrients and minerals such as calcium (ie. lactose intolerance), food aversion, bowel symptoms, and/or possible altered absorption. Fibrostenotic CD is characterized by debilitating strictures where patients are known to alter food intake to avoid obstructive symptoms. However, relationships between food intake patterns and BMD have not been well delineated according to CD phenotypes.

**Purpose:**

Our study evaluated 1) BMD as measured by dual X-ray absorptiometry (DXA), and 2) energy intake, dietary components and/or micronutrients in CD patients with strictures versus inflammatory (non-stricture) behaviour.

**Method:**

In this prospective pilot study, patients > 55 years old with ileal CD strictures or inflammatory behavior were recruited from the University of Calgary IBD clinic. All patients completed hip and spine DXA scans and two dietary assessment questionnaires: 1) The Automated Self-Administered 24-hour Dietary Assessment Tool (ASA24) and 2) the Diet History Questionnaire III (DHQ III). Additional data collected included past fracture history, medication (glucocorticoid exposure), smoking, and surgical history. Standard of care laboratory investigations obtained included C-reactive protein, parathyroid hormone, calcium, albumin, and 25-hydroxyvitamin D. Patients with celiac disease, cirrhosis, heart failure, kidney disease, short gut, estrogen use, and dysphagia were excluded. Independent samples t-test and multi-variable regression analyses was conducted.

**Result(s):**

Seventeen patients had stricturing and twelve had non-stricturing CD (demographics Table 1). The mean BMD for non-stricturing CD patients was not significantly different from those with a stricturing CD phenotype (p =0.140). Non-stricture patients consumed significantly more dairy, calcium, and phosphate. For all CD patients, there was a positive correlation with BMD and intake of fat (p=0.03), carbohydrates (p=0.01), fiber (p=0.01), and alcohol (p=0.01). There was no statistically significant difference in corticosteroid exposure or smoking status. 74.7% (11/17) patients with stricturing CD had past bowel resection compared to only one patient with non-stricturing CD.

**Image:**

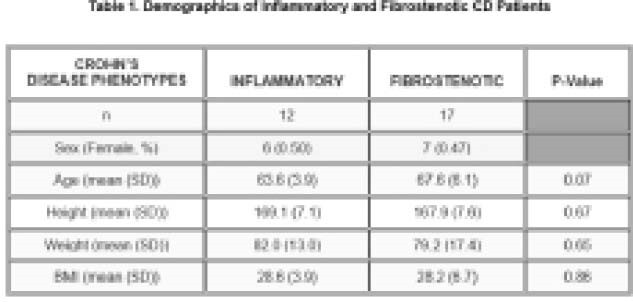

**Conclusion(s):**

In this pilot study, there was no difference in BMD between CD patients with and without small bowel strictures despite inflammatory behaviour patients having less surgical resections and consuming more calcium rich foods known to improve BMD. Further studies may delineate the dietary differences among CD phenotypes and provide information for interventions for nutrient supplementation, and a greater understanding of their relationships with BMD.

**Please acknowledge all funding agencies by checking the applicable boxes below:**

Other

**Please indicate your source of funding;:**

Koopmans Memorial Research Fund

**Disclosure of Interest:**

None Declared

